# Group or individual lifestyle-integrated functional exercise (LiFE)? A qualitative analysis of acceptability

**DOI:** 10.1186/s12877-020-01991-0

**Published:** 2021-02-01

**Authors:** Leah Reicherzer, Franziska Kramer-Gmeiner, Sarah Labudek, Carl-Philipp Jansen, Corinna Nerz, Malin J. Nystrand, Clemens Becker, Lindy Clemson, Michael Schwenk

**Affiliations:** 1 School of Public Health and Community Medicine, University of Gothenburg, Gothenburg, Sweden; 2 Network Aging Research (NAR), Heidelberg University, Heidelberg, Germany; 3 Department of Clinical Gerontology and Geriatric Rehabilitation, Robert-Bosch-Hospital, Stuttgart, Germany; 4 Faculty of Medicine and Health Sciences, University of Sydney, Sydney, Australia

**Keywords:** Fall prevention, Focus groups, Qualitative methods, Lifestyle-integrated exercise, Habit formation, Group vs individual exercise, Qualitative content analysis

## Abstract

**Background:**

The Lifestyle-integrated Functional Exercise (LiFE) program is an effective but resource-intensive fall prevention program delivered one-to-one in participants’ homes. A recently developed group-based LiFE (gLiFE) could enhance large-scale implementability and decrease resource intensity. The aim of this qualitative focus group study is to compare participants’ experiences regarding acceptability of gLiFE vs LiFE.

**Methods:**

Programs were delivered in seven group sessions (gLiFE) or seven individual home visits (LiFE) within a multi-center, randomized non-inferiority trial. Four structured focus group discussions (90–100 min duration; one per format and study site) on content, structure, and subjective effects of gLiFE and LiFE were conducted. Qualitative content analysis using the method of inductive category formation by Mayring was applied for data analysis. Coding was managed using NVivo.

**Results:**

In both formats, participants (*N* = 30, 22 women, n_gLiFE_ = 15, n_LiFE_ = 15, mean age 78.8 ± 6.6 years) were positive about content, structure, and support received by trainers. Participants reflected on advantages of both formats: the social aspects of learning the program in a peer group (gLiFE), and benefits of learning the program at home (LiFE). In gLiFE, some difficulties with the implementation of activities were reported. In both formats, the majority of participants reported positive outcomes and successful implementation of new movement habits.

**Conclusion:**

This is the first study to examine participants’ views on and experiences with gLiFE and LiFE, revealing strengths and limitations of both formats that can be used for program refinement. Both formats were highly acceptable to participants, suggesting that gLiFE may have similar potential to be adopted by adults aged 70 years and older compared to LiFE.

**Trial registration:**

ClinicalTrials.gov, NCT03462654. Registered on March 12, 2018.

**Supplementary Information:**

The online version contains supplementary material available at 10.1186/s12877-020-01991-0.

## Background

The incidence of falls in older persons is expected to increase in upcoming years [1]. Widely disseminated fall prevention programs for community-dwelling, fall-prone older adults aim to impede falls and their individual and socio-economic consequences.

Most evidence-based fall prevention programs are based on ‘structured’ group exercises conducted at least once a week [2, 3]. These programs often fail to sustain long-term effectiveness due to regressive adherence rates [4].

The alternative approach of lifestyle-integrated training [5] aims for higher adherence rates through long-term behavior change. The Lifestyle-integrated Functional Exercise (LiFE) program [6] embeds functional strength and balance activities into daily life to enhance physical function and activity of adults aged 70 years and older. Implementing activities in recurrent opportunities is used as a strategy to help participants create new habits [7], which constitutes a key mechanism for long-term maintenance of behavior change. LiFE recorded a significant reduction in fall rate (31%) compared to the control group (gentle and flexibility exercises). According to a recent review [8], adherence for LiFE was higher compared to structured training during the intervention periods. One randomized controlled trial rated completing LiFE activities on at least three days a week, or structured home exercises three times a week, as 100% adherence [6]. The results showed significantly higher adherence to LiFE (64% of participants) compared with structured training (53%) [6]. Poor adherence (<25%) was evident in 19% of structured training compared to 7% of LiFE participants [6].

However, its delivery through seven one-to-one home visits requires considerable time and human resources. This has recently been addressed by Kramer and colleagues [9], who developed and tested a potentially resource-saving group-based LiFE (gLiFE) concept, delivered to eight to twelve participants by two trainers in seven group sessions. The authors reported successful implementation and regular execution of activities in daily life: after the last intervention session, participants (*n* = 6) had implemented 9.5 (IQR = 4.0) out of 14 LiFE activities into their daily lives. Furthermore, five out of six participants had implemented activities five days a week over the course of the program. Effectiveness, however, has not been established yet, as gLiFE is currently being evaluated in an ongoing trial in comparison to the individual LiFE program [10].

Besides evaluating intervention effectiveness, considering participants’ attitudes towards such interventions and whether they are acceptable, is essential for long-term success [11]. Acceptability is related to the perceived appropriateness based on anticipated or experienced cognitive and emotional responses to an intervention [12]. The construct of acceptability encompasses different components: affective attitudes, burden, ethicality, intervention coherence, opportunity costs, perceived effectiveness, and self-efficacy [12]. Levels of acceptability may critically affect how likely participants are to adhere to home-based exercise, and consequently to benefit from the intervention [13]. Conversely, low acceptance of the intervention can negatively impact its effectiveness [14]. Assessing LiFE's and gLiFE's acceptability is indispensable for intervention refinement, and vital for both research and clinical practice to understand how the intervention formats can be sustainable over time. To date, only a few qualitative studies on this topic exist, although a thorough analysis is recommended in the evaluation of complex intervention by the Medical Research Council guidelines [15]. The main differences between gLiFE and LiFE that may influence participants’ acceptance of the intervention are the following:

LiFE participants receive the program directly in their home, facilitating identification and testing of suitable daily situations for implementing LiFE. One-to-one delivery allows a highly personalized training and closer contact between trainer and participants, which could influence participants’ exercise behavior and perception of the program. A previous qualitative study on a different LiFE approach [8] for adults age 60 to 70 suggested that trainer support was a strong motivator to carry out the activities. Participants valued the flexibility and personalized nature of the program. In gLiFE, different strategies were implemented to compensate for these missing aspects of individual delivery.

gLiFE participants can potentially benefit from learning the activities in a group. A previous group-based LiFE study [16] suggested that participants valued the support from and interaction within the group. Fellowship and shared experiences with peers have been described as facilitators in maintaining long-term exercise [17]. Social support from an exercise group can enhance motivation [18], affective responses, and the benefits of the intervention [19, 20].

Regarding a potential large-scale dissemination of gLiFE, the current study aims to explore how experiences of participation differ between gLiFE and LiFE, and whether both formats are acceptable to the target population of fall-prone older adults, aged 70 years and older.

## Methods

### Study design and setting

This qualitative study was conducted with a subsample of participants of the LiFE-is-LiFE trial [10], a multi-center, single-blinded, randomized non-inferiority trial comparing gLiFE with LiFE regarding fall reduction and cost-effectiveness (ClinicalTrials.gov, NCT03462654) (period: 06/2018 to 08/2020). Participants with a verified fall risk took part in either seven gLiFE or LiFE sessions within eleven weeks, followed by two phone calls. Follow up assessments were performed after six and twelve months.

For the present study, qualitative data collected in four focus group discussions after the six-month follow-up assessments (04/2019) were analyzed.

### Programs

LiFE aims to reduce fall-related outcomes and promote long-term physical activity in community-dwelling older adults by integrating balance and strength activities into their daily routines. Program details of gLiFE [9] and LiFE [21] are described elsewhere. Table 1 provides an overview of the similarities and differences between both formats.

**Table 1:** Similarities and differences between LiFE and gLiFE conducted in the LiFE-is-LiFE trial [10]

	LiFE	gLiFE
Brief aim	Improve balance and lower limb strength, increase physical activity, decrease risk of falling, long-term sustainability of the LiFE activities through habit formation and self-empowerment	
What: Materials	Participant’s manual, German version [22]; Contains descriptions and instructions of LiFE activities; principles of balance and strength training as well as physical activity enhancement; safety instructions when performing the activities; background on balance and strength exercise; assistance and support for changing habits and performing LiFE activities Trainer’s manual, German version; one for LiFE, one for gLiFE. Contains all information also included in the participant’s manual; additionally: outline of all 7 sessions and 2 phone calls, including text templates, material, preparations, and precautions Workbook; for all participants; used during intervention: Includes information on study procedures, personnel, contacts, and safety instructions; activity planning sheets for balance, strength, and physical activity; activity counter, notes pages; LiFE principles Aids and materials during intervention sessions: Laminated cards, showing LiFE principles and LiFE activities to be used as visual aids during intervention sessions; balls, blankets, sponge rubber, boxes, clipboards, pens, bags, name tags, flipcharts	
What: Procedures	7 home visits by one qualified trainer; 2 phone calls 4 and 10 weeks after last session	7 group sessions (*n* = 8–12 participants) led by one main and one co-trainer, 2 phone calls 4 and 10 weeks after last session
Who provided	Trainers are sport scientists, physiotherapists, occupational therapists or psychologists. All trainers received a two-day training course on the program background, aims, and components prior to the project start.	
How	One-to-one situation in the participant’s home	Group setting with 8–12 participants and two trainers
Where	Two study sites: Heidelberg and Stuttgart (Germany)	
When and how much	7 sessions within 11 weeks: week 1, 2, 3, 5, 7, 9, 11. Two phone calls 4 and 10 weeks after the last session (i.e. week 15 and 21). Duration of each session: 1–1.5 h	See LiFE Duration of each session: 2–2.5 h
Setting	Intensity and dose are determined by the individuals’ activity plans, adherence, and performance level of each activity	
Behavior change	Behavior change theories based on LiFE trainer’s manual and participant’s manual [21].	Modification of the original behavior change concept using established theories on health behavior, such as the Health Action Process Approach [23, 24] and the Self-Determination Theory [25]. Intervention contents of gLiFE were mapped using the Behavior Change Technique (BCT) Taxonomy v1 [26].

Program sessions of both formats were administered by physiotherapists, occupational therapists, and/or sport scientists that had attended a two-day workshop to ensure standardized delivery. Trainers teach the participants how to perform the activities (e.g., squat), where (e.g., in the bedroom), and when to implement these into daily routines (e.g., each time when reaching for the floor drawer). New movement habits are created by linking the LiFE activities to specific daily situations based on behavior change concepts [9, 27].

Participants learn how to adapt chosen activities to their lifestyle and how to increase difficulty to ensure continued progression using LiFE principles [21].

The main difference between programs is the delivery format: group delivery for eight to twelve participants by two trainers (gLiFE) compared to one-to-one delivery in participants’ homes (LiFE). In gLiFE, the trainer’s role is to teach and facilitate; in LiFE, the trainer teaches and substitutes a training partner. Contents of gLiFE and LiFE are taught in predefined order, but teaching in gLiFE is organized in an interactive manner including group discussions and joint activity practice with peers.

All participants receive the German participant’s manual [22] and a workbook, including a modified activity planner [9] and an activity counter to plan and monitor activity performance.

### Participants

A total of 310 community-dwelling older adults (> 70 years) were randomized to either gLiFE or LiFE at two study centers in Germany (Network Aging Research, Heidelberg University; Robert-Bosch-Hospital, Stuttgart). For this study, 30 participants (22 women, 8 men; *M*_*age*_ = 78.8; range 70–96 years; n_gLiFE_ = 15; n_LiFE_ = 15) were purposively selected [28] from the trial. We ensured to include participants who reported higher and lower levels of habitualization of the LiFE activities at the six-month follow-up. Habitualization was indicated by the Self-Report Behavioural Automaticity Index (SRBAI) [29]. The SRBAI median split [30] was defined as the threshold (SRBAI ≥4.49 = higher behavioral automaticity, SRBAI ≤4.49 = lower behavioral automaticity). We composed each focus group of participants with different ages, gender, and different SRBAI scores to maximize the breadth of information and foster discussion between participants that did and did not successfully implement LiFE. To determine whether the sample size of our study and the number of focus groups conducted was sufficient, we followed the systematic model of information power in qualitative study [31]. It states that a study will need a smaller number of participants if the study aim is narrow, if the combination of participants is very specific for the study aim, and if the study is supported by established theory. Taking into consideration that these aspects are fulfilled in this study, in combination with empirical evidence by Guest et al. [32] identifying that 90% of all themes found in a focus group analysis were discoverable within 3 to 6 groups, we determined that the number of focus groups and participants is sufficient to provide the information we sought.

**Fig. 1:**
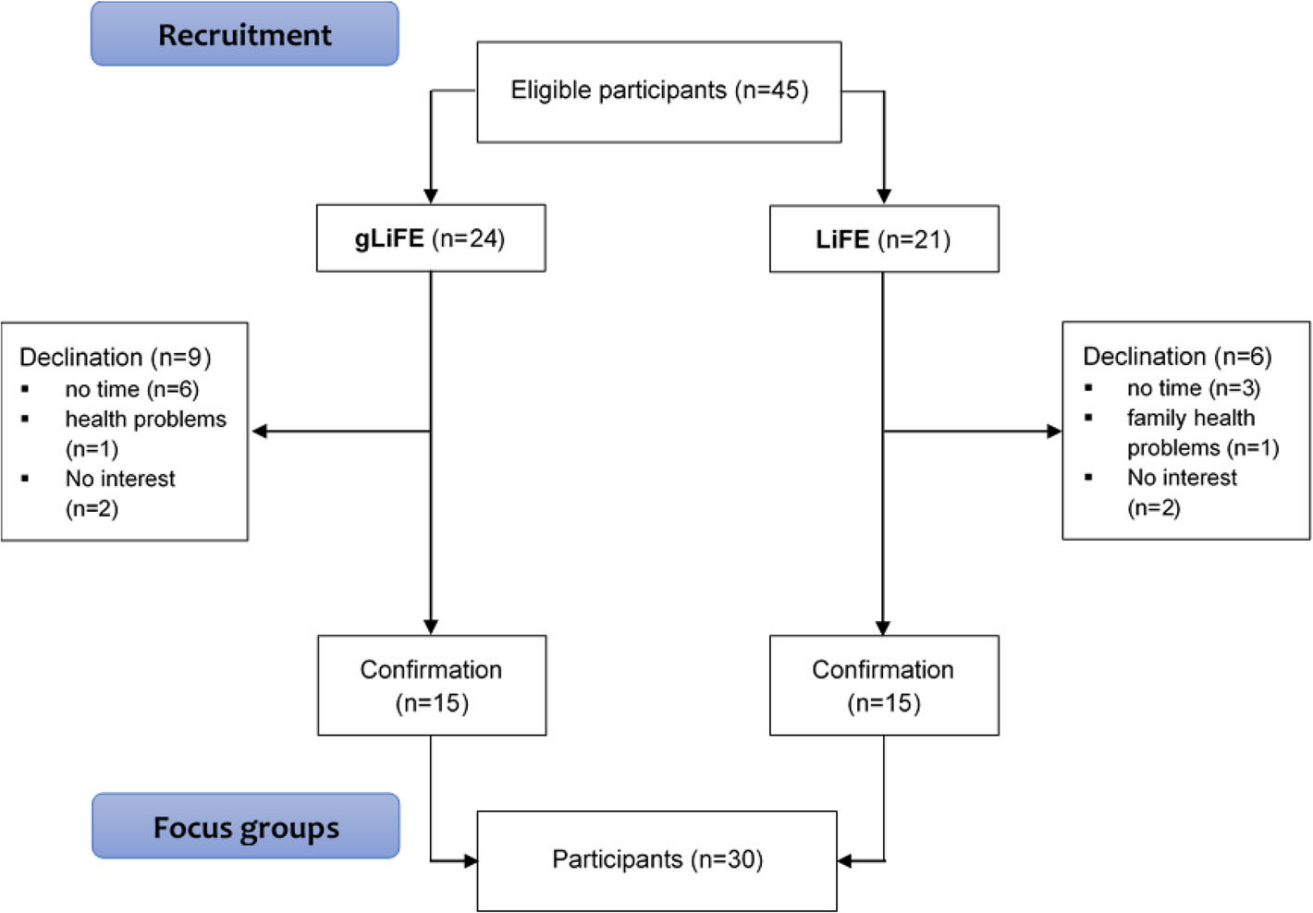
Recruitment process

Participant characteristics are summarized in Table 2; they were predominantly female (73%), educated, at risk of falling indicated by the Timed Up and Go Test [34], and were cognitively healthy according to the Montreal Cognitive Assessment (MoCA) [33]. On average, participants had two co-morbidities and took five medications per day. Participants were invited via telephone. The recruitment process is shown in Fig. 1.

**Table 2:** Characteristics of participants (*N* = 30)

	Mean (SD) or % (n)		
	Total (***N*** = 30)	gLiFE (***N*** = 15)	LiFE (***N*** = 15)
Age, years	78.8 (6.6)	78.5 (6.1)	79.1 (7.2)
Women	73.3 (22)	73.3 (11)	73.3 (11)
MoCA, score (0–30)	26.0 (1.7)	26.5 (1.8)	25.4 (1.5)
Fall incidents at baseline	30 (9)	20 (3)	40 (6)
Completed years of education	14.1 (3.8)	14.5 (4.5)	13.7 (3.0)
Highest school degree			
Secondary school	43.4 (13)	46.7 (7)	40 (6)
Advanced college certificate	3.3 (1)	–	6.7 (1)
General university entrance qualification	50 (15)	46.6 (7)	53.3 (8)
No degree	3.3 (1)	6.7 (1)	–
TUG, sec.	12.7 (3.4)	12.7 (2.7)	12.7 (4.0)
BMI	27.3 (4.6)	26.6 (4.5)	28 (4.7)
Co-morbidities, number (0–6)	2.3 (1.5)	2.5 (1.6)	2.2 (1.4)
Medications, number (0–21)	5.2 (4.2)	5.4 (4.6)	5.1 (3.8)

*Not*e. *MoCA* Montral Cognitive Assessment [33], *Fall incident*s Falls in the last 6 months at baseline measurement, *TUG* Timed Up and Go Test [34], *BMI* Body Mass Index

### Data collection

An interdisciplinary team of exercise scientists, psychologists, and physiotherapists developed a semi-structured interview guide (see Additional file 1) for both formats based on a previous LiFE focus group discussion [9].

The interview guide comprised 14 main questions on the programs and their components, the group and individual format, and habit formation processes like action planning or building new movement habits. It was piloted with one LiFE-is-LiFE participant regarding clarity of questions and refined after pilot evaluation.

Two focus group discussions were conducted at seminar rooms in each study center, one for gLiFE (n_Stuttgart_ = 8, n_Heidelberg_ = 7), one for LiFE (n_Stuttgart_ = 7, n_Heidelberg_ = 8) lasting between 90 and 100 min. At the beginning, the study purpose (evaluation of program acceptability) was explained, and participants gave written informed consent. The moderator facilitated discussions by asking questions with follow-up prompts, probing, encouraging reserved participants to speak, and ensuring that discussions covered the main topics. The moderator (main author, physiotherapist, and external researcher) and co-moderators (team members) were not involved in follow-up assessments and intervention delivery. Co-moderators took notes and kept time. Two of the co-moderators who took part in the program development stayed silent during discussions. Focus groups were audio recorded and transcribed verbatim in German, according to transcription guidelines by Kuckartz [35].

### Data analysis

We performed a qualitative content analysis according to Mayring [36], following the procedure of inductive category formation. Coding was managed using NVivo 12 (QRS International, Australia). Categories were not predetermined but instead defined by the researchers as near as possible to the material [36]. The step of paraphrasing all text can be skipped and only the material relevant for a specific research question has to be considered. For this selection process, we formulated a selection criterion including the definition and components of acceptability [12] and more general evaluations of the programs. This approach was chosen to be open to themes emerging from participants’ statements that may not directly relate to acceptability (e.g. on habit formation), but are nevertheless important to understand how to refine the program.

All focus group discussions were defined as the unit of analysis and the manifest content was analyzed. The selection criterion (expression of affective attitudes towards the programs, burden, coherence, perceived effectiveness and self-efficacy, and general evaluations of program components of the LiFE or gLiFE program) was used to determine the relevant material from the text. In addition, a level of abstraction was determined, which defines how general or specific categories should be formulated [36]. Based on this, text was coded line-by-line and either a category was constructed and named every time an element of the text fulfilled the selection criterion (see Table 3), or the text was subsumed under an existing category. After 50% of the text, categories and coding rules were revised, then two authors (LR, FKG) independently coded the text. Main categories were formulated and discussed. In case of a disagreement, a third researcher (SL) was consulted. Finally, categories were organized into overarching themes and contents were contrasted by group (gLiFE vs LiFE). The organization and naming of overarching themes was shaped by the main topics of the interview guide. Three authors (LR, FKG, SL) agreed on the final category framework.

**Table 3:** Example of inductive category formation

Coding unit	Keywords	Category	Main category
*“I would have liked to go to a group. I found the individual home visits, it was a bit too less. And a group is more intense”.*	Group is more intense	Motivation because of group	Format of the program
*“No, the group pressure is necessary. For me at least. […] Always just my own enthusiasm, it is limited”.*	Group pressure is helpful		

## Results

Five overarching themes were identified: Program overall, trainer support, content of the program, format of the program, and changing behavior. The results are illustrated by quotes, translated from German to English. Participants are identified by group, gender, and age (e.g., gF73 = gLiFE, female, 73 years; oM80 = original LiFE, male, 80 years).

### Program overall

Participants reaction to the overall program did not differ much in the two program formats. In both formats, participants were positive about the program: *“The LiFE-program is great and I enjoyed it”* (gF73*); “It seemed [ …]* *very well structured”* (oM78). Most participants understood and liked the concept of lifestyle-integrated exercise: *“And what I found appealing is that these are exercises that can be integrated into daily life”* (gF70). Many participants further valued the focus on independent exercising: “*Doing my own thing at home alone, not having to join any sports clubs or groups, that is exactly the right thing for me”* (oM80).

Participants experienced some troubles with the paperwork for the study. The monthly fall calendar and the set of questions at baseline and follow-up assessments were perceived as “*quite annoying” (*gF70) by some participants in both formats.

### Trainer support

In general, gLiFE and LiFE participants spoke positively about the teaching styles of their trainers: *“The guidance and instructions were great [ …] and well explained”* (gM82) and “*I think, my impression was entirely positive. He [the trainer] had a very good pedagogical approach. So, it was very clear*” (oM80).

Participants in both formats felt individually supported by the trainers during teaching sessions. LiFE participants described how the trainers adapted the programs to their abilities and gave feedback on their performance during activities: *“She really catered to my needs and abilities and had another idea, on how to adapt things if I could not do them” (oF72).* gLiFE participants described how the trainers approached them individually within the group setting. *“They really responded to the individual’s situation” (gF77); “They would correct the execution of activities in a very caring way, I would say. So, when someone did not do it correctly, then they very gently approached you and said, try this or try that.” (gF70).*

For gLiFE participants, it seemed important not to feel pressured by trainers or exposed in front of their peers when they were having difficulties with an exercise: *“You never had to feel embarrassed. For example, I have a problem with my hip and I cannot step over objects sideways. But nobody gave me a weird look and I could just tell the trainers that I cannot do it […] and never felt pressured”* (gF72).

LiFE focus groups discussed their relationship with the trainers in more detail, for example by praising their personality (e.g. how cheerful or friendly they were): *“He [the trainer] always arrived with a big smile on his face and we were always happy to see each other. He was always so cheerful, even in the morning”* (oF80). Furthermore, they described the one-on-one supervision as an opportunity for a personal exchange with the trainer: *“So I liked that you could talk to them about personal stuff, too. There was an exchange and I really enjoyed that. We had great conversations”* (oF74).

### Content of the program

#### Structure and materials

Participants from both formats liked the *“whole structure”* (gF84) of sessions and the *“well balanced and instructive*” (gM82) combination of theory and practice. One gLiFE participant specified that the repetition of activities from the last sessions was helpful: “*It really gets stuck in your mind; you don’t hear it just once and then you have to be able to do it and then there’s a different program next time, but instead it was repeated before adding something new*” (gF80).

In both formats, participants valued the manual as a helpful tool “*especially at the time when the trainer is not there anymore”* (oM74).

#### Activities

gLiFE and LiFE participants indicated strong preferences for activities that are easy to integrate into daily routines, like the one-leg stand: *“I think the most beneficial are the activities, that can be successfully implemented in everyday tasks” (*oM80)*.* Participants of both formats talked about activities which were difficult to perform or which they perceived as *“not natural”* (gF73) or *“silly”* (oM80), like “*stepping over objects backwards”* (gF91). They often related difficulties with certain activities to personal health conditions or pain:“*For me it is difficult, because my knees cause a lot of troubles. I cannot do many of the activities”* (oF96).

gLiFE participants reported that they consider safety aspects when practicing at home: *“I always make sure, when practicing [ …*], *that I am close to the wall”* (gF82). LiFE participants did not make specific statements on safety.

Only gLiFE participants suggested to add more activities, like “*some kind of coordination”* (gF84) or to practice more complex movements like *“getting out of a bathtub”* (gF82). One LiFE participant wished for more specific fall prevention exercises to learn *“how to fall and how to compensate a fall” (oF73).*

#### Intensity and duration

Participants had different opinions on program intensity, with most being satisfied. Few expressed that the intensity *“was slightly too little”* (oF80) compared to similar exercise programs. Some participants wished for more practice time during sessions, as well as increased difficulty or more challenging exercises: “*Strength could be a little bit more [challenging], to guarantee stability”* (oM83).

### Format of the program

#### Group format

When asked about their thoughts on the other format, gLiFE and LiFE participants addressed advantages of group exercising. gLiFE participants reported that the group enhanced their motivation: “[ …] *it wakes your ambition. You do not want to step down, you want to keep up with the others”* (gF88). A good atmosphere in their groups motivated and encouraged them: “[ …] *in the group it is, I think, a bit funny from time to time. You encourage each other”* (gF88). Not all gLiFE participants had the same experience of group cohesion in their groups and had wished for more group interaction: “*So, to be honest, I never felt a sense of companionship, unfortunately. ( …*) *I would have liked to experience some team spirit, to have an exchange”* (gF84).

Several gLiFE participants described the exchange and comparison with peers as “*comforting*” (gF91) because they “*all face difficulties with walking and climbing stairs, and they are all troubled by their knee pain”* (gF91).

LiFE focus groups discussed “group pressure” and peer exchange as positive effects of exercise groups, based on previous experiences or preconceptions: “*The group pressure, [ …] yields more than fumbling around alone with the trainer”* (oM74). Some said they would have preferred “*being part of a group of like-minded people”* (oF96) for the social aspects, and to have an exchange with peers because *“you always have this one person [the trainer], who you of course can always have an exchange with, and who gives good advice, but in everyday life it’s different. We’re all older people and you [research team] can’t really understand how we feel” (oF82).*

#### Individual format

LiFE participants agreed that it was helpful to receive individual support to identify situations and locations suitable for the implementation of activities directly in one’s home: *“The advantage of him [trainer] being in my home was that we could choose situations together in which it [activity] can be implemented”* (oM80). LiFE participants appreciated the flexibility of home visits (e.g. individual scheduling, no travel time) and the individual supervision by a trainer: “*That’s why I was really glad to have my own trainer. Who could tell me, that I was doing it correctly. Who corrected me”* (oF80).

gLiFE participants suggested that receiving one home visit in addition to the group sessions, *“to have one’s attention directly drawn to where in the house, when in the household, you could do this”,* would be *“an enhancement”* (gF84).

In LiFE, the transition from being supervised by a trainer at home to practicing alone might have been more difficult; one LiFE participant recalled the two booster phone calls as important: “*First you have the regular supervision and suddenly it stops. And then you have to see how to get on alone. And I found it [phone call] quite good”* (oF72).

### Changing behavior

#### Forming habits

Participants from all focus groups identified opportunities to integrate activities into daily routines, and some activities became habitual: “*For me, it became a habit – I don’t want to say that I do everything. But now I tend to remember it and then I do it” (gF82)* and *“[ …] it’s like learning a new language. In the beginning you’re studying two, three hours every day and then at one point you know the basics and can just use them without thinking”* (oM78). gLiFE and LiFE participants described activities being connected to situational, object-related, or activity-based cues: “*It did indeed remind me, when in a certain course of action, AHA!, now you could integrate this”* (gF84).

#### Planning actions

One key psychological strategy of both interventions is action planning. One gLiFE participant described that the activity planning helped her: “*It [activity planner] was really good to get started [ …] because it provides an incentive [ …] to actually do it*” (gF70). However, a few gLiFE participants felt like the action plans they made during the group sessions were not applicable in a home environment: “*How I was doing it in the beginning, doing this and that while brushing teeth, that just did not work” (gF78)*. Some LiFE participants found the activity planner tedious and *“too silly*” (oM80). One LiFE participant specifically stated*: “I would like to do it spontaneously [ …] I could never say, so now when I brush my teeth I do this forward and backward thing, only when it comes to my mind I do it”* (oF82).

#### Outcome experiences

The majority of gLiFE and LiFE participants shared positive outcome experiences, like improvements in physical function or mobility (“*Since I walk the stairs so often, [ …] my knee became better”*, gF70) or a more active daily life (“*I use the car less often”*, oF74). A few participants reported improvements in their fear of falling: “*For me it took away the fear of falling” (*oF92). Others stated that their fear of falling was persistent: *“Despite participating in this program, I am always afraid of falling again”* (gF78).

#### Confidence in doing

gLiFE and LiFE participants were confident about their capability of performing the activities and saw practicing LiFE as their own responsibility: “*If I don’t have the discipline myself, then another session is not going to help me [ …*]” (oF72). A few participants in gLiFE reported adopting a role as a motivator for others to be more physically active: “*So you can teach others, if you want to of course and if they accept it. You can motivate them and say, simply integrate this into your daily life. And that does, it does really help”* (oF82). Some LiFE participants anticipated that their exercise routine might fade without regular home visits over time. One of the LiFE focus group participant stated that not seeing the personal relevance for themselves, or more important things on their agenda, kept them from implementing activities into their lives: “*As I have already said, I don’t take this [LiFE] so seriously. I have a lot of other things to do in my life, I have a house, I have a garden to take care of and then I also have a lot of hobbies* (oF80)”. Both LiFE participants and gLiFE participants describe their confidence being influenced by what others might think about them exercising: “*I live on the ground floor and many people pass by outside and if I for example walk on my heels I watch and make sure that nobody thinks, ‘oh god, what is she doing, how is she walking around so stupidly.’ It looks so foolish”* (oF74).

## Discussion

This is the first qualitative study comparing and describing participants’ experiences of gLiFE and LiFE, to identify whether both programs are acceptable to community-dwelling older adults at risk of falling. The programs’ acceptability to the participants will be analyzed using a selection of Sekhon’s [12] component constructs for acceptability, namely: intervention coherence, affective attitudes, burden, perceived effectiveness, and self-efficacy.

Participants found both LiFE programs acceptable, indicating that both formats are suitable for the target group. LiFE’s main aim, integrating the activities into daily life, was well received and understood by gLiFE and LiFE participants. This perception of so-called intervention coherence (the extent to which participants understand the goal of an intervention and the mechanism behind it) positively influences acceptability [12]. The possibility to train independently in one’s home was perceived as a strength of both LiFE formats. These results underline findings from previous LiFE feasibility studies delivered one-to-one [6, 8] or in a group [9, 16, 37], further supporting that LiFE can be seen as a promising alternative to structured fall prevention programs [5], also in a group setting.

In both formats, the support by trainers played an important role for the participants’ affective attitudes towards the program. Previous studies highlighted that professional help and the motivational support of an exercise specialist were important factors in older adults’ attitudes towards, and attendance of, exercise classes [38]. The perception of being addressed individually by trainers, in both LiFE and gLiFE, indicates that gLiFE also offers opportunities for individual support. Our findings are in line with a previous study of LiFE showing that individual content adaptation is indispensable to enhance acceptability and exercise adherence [39]. Only LiFE participants addressed their trainer’s personality traits, suggesting that individual training and personal exchange strengthen the trainer-participant relationship in LiFE compared to gLiFE. gLiFE participants appreciated that they never felt pressured by trainers when they had difficulties performing some LiFE activities during group sessions. This is in line with research showing that a trainer’s controlling coaching style can decrease a participant’s autonomous motivation [40]. Trainers that reaffirm participants in their own decision-making and respect their individual capabilities are an important factor for building motivation and confidence in gLiFE. The perception of self-efficacy, i.e. the participants confidence to perform a required behavior, was found to be crucial for the acceptability of an intervention [12]. In gLiFE and LiFE, the majority of participants said they were confident in being able to perform the activities and to maintain the exercise routine. Our findings suggest that both formats were able to support participants in building the confidence to sustainably engage in LiFE.

The structures of gLiFE and LiFE were well received, particularly the repetition of learned activities at the beginning of each group session were perceived as helpful to remember and to consolidate the movement execution. This indicates that the structural modifications developed for gLiFE [9] were appropriate. As in previous LiFE studies [9, 41] participants perceived the LiFE manual as a helpful tool for corrections which highlights usefulness of a manual in both groups.

In both formats, participants preferred activities that were easy to integrate in daily life. The importance of activities being achievable and easy to integrate into daily life routines has been discussed before [41]. Specific LiFE activities were perceived as too artificial or difficult to perform, e.g. due to personal health problems. This finding has already been described by Boulton et al. [8]. Therefore, assessing which and why certain activities are not feasible, as well as increasing participants' autonomy might be essential. Indeed, previous qualitative evaluations of LiFE showed that integrating participants’ ideas may be important to facilitate their long-term goal achievement [8].

gLiFE participants stated that they took care of their safety while practicing LiFE at home, so gLiFE seems to sufficiently convey important safety aspects of home exercise. We can only speculate why LiFE participants did not discuss safety aspects: maybe these were considered less important or handled more naturally without participants actively reflecting on them as they learn and practice LiFE directly in their home setting. Fear of falling, and improvements in fear of falling, were mainly addressed by LiFE participants. Addressing the fear of falling was not in the scope of the programs, and this focus of participants could be explained by their previous experiences: LiFE participants in our sample reported a higher number of fall incidents at baseline compared to gLiFE participants.

The burden of participation is a relevant component construct of acceptability [12]. The required paperwork for the study (e.g. monthly fall calendar) was perceived as time-consuming by both gLiFE and LiFE participants. Even though documentation mainly related to the study and will be much shorter in a potential roll-out of the programs, it may have influenced acceptability of programs. Overall, participants statements indicate satisfaction with gLiFE’s and LiFE’s intensity, suggesting that the perceived effort to participate in the program (i.e. physical and cognitive requirements) was generally appropriate. A few participants of both formats felt they were not challenged enough by the activities after some time. The principle of upgrading the difficulty of the activities might have been insufficiently conveyed by the trainers, therefore the participants may not have fully understood that they should adapt activity intensity. On the other hand, it could be that participants preferred to practice in their “comfort zone”, instead of putting themselves into unstable or exhaustive situations to challenge themselves. Revising the theory class on upgrading exercise should be considered for future studies to ensure the participants’ awareness of the importance of gradual intensity progression [42]. The nature of lifestyle-integrated exercise (small activity bouts throughout the day) might feel less challenging compared to exercising for a set period of time.

Participants of both formats named various benefits when asked about their thoughts on learning LiFE in groups (e.g. being more motivated due to social comparison or social support), which is in line with studies that emphasize the importance of peer contact in fostering positive physical activity experiences among older adults [43, 44]. Studies demonstrate that when people do exercise in groups, especially when participants experience cohesion, adherence levels [19] or outcomes like functional balance [45] improve significantly compared to exercising alone. As argued in the Self-Determination Theory [25], social contextual events like peer feedback can foster feelings of competence and enhance intrinsic motivation. Low motivation has been identified as a cause for adults not to adhere to home-based activities [18]. Based on our findings, the social aspects of the group as a motivating factor could be considered an advantage of gLiFE over LiFE. Nonetheless, not all gLiFE participants experienced group cohesiveness. Previous studies showed that task and social cohesion (individual attraction towards the group task and the group members) are related to older adults’ exercise adherence [46]. We suggest that the facilitation of group processes in gLiFE should be refined to foster group cohesion. Increasing the feeling of being understood by the trainers could be achieved by employing trainers which are nearly the same age as participants. This has been found effective in other settings like diabetes care [47].

LiFE participants were satisfied about receiving individual training at home. This supports previous research [48] pointing at the preference of older adults’ for home-based activities and/or exercising alone, and highlights the importance of individual preferences for exercise settings. gLiFE could be a good compromise as it combines group-based teaching with independent home-based training.

A single home visit to support implementation of activities was suggested as a possible improvement to gLiFE by participants. Adding one single home visit to gLiFE would reduce its’ assumed low-cost delivery, and hence financial feasibility and large-scale implementability from a stakeholder perspective. Although some gLiFE participants had difficulties finding the right daily cue during group sessions, results suggest that the principle of tying LiFE activities to different situational cues in order to create new movement habits was understood by most participants. This supports study findings of group-based [16] and individual LiFE [8]. The fact that not being in the home environment could cause difficulties in action planning was addressed in the design of gLiFE by including compensational strategies, like group discussions to collect the participants’ suggestions for possible situations to implement activities [9]. Not only in gLiFE did difficulties in action planning occur, as LiFE participants also perceived action planning and habit formation as challenging. In the future, more guidance and direct suggestions from trainers should be offered for action planning if needed.

Participants from both LiFE formats spoke about perceiving positive program effects, like more activity in daily life, and improvements in mobility and function. Perceived effectiveness has been described as one relevant property of acceptability [12]. Positive outcome experiences were found to increase satisfaction, which increased the likelihood of a sustained exercise routine [49].

To summarize, we describe the important differences between both intervention formats: in gLiFE, the social aspects of learning the program in a peer group seem to positively influence participants’ affective attitudes towards and motivation to participate in the intervention. Indeed, some LiFE participants wished to take part in the group to benefit from social interactions and new peer contacts after the program ended. On the other side, LiFE participants appreciated the one-to-one training and valued the individual training as an advantage for implementing the LiFE activities into daily routines. In gLiFE, the implementation of activities into daily routines was perceived as more difficult compared to LiFE. Regarding acceptability, individual training may decrease the burden placed on participants: less travelling is needed, sessions can be scheduled flexibly, and the supervision is individual.

Our analysis identified several similarities between both intervention formats: Participants reported a positive overall attitude towards the programs, and specific program features like the selection of activities. The majority perceived the program as having effects on their daily life activity or movement habits and were confident in their ability to keep practicing LiFE (self-efficacy). Overall, this focus group study indicated that both LiFE formats were acceptable to the participants.

### Strengths and limitations

Qualitative methods play a valuable role in exploring participants’ experiences of study participation. Their use is increasingly recognized as the best practice in the development [15] and evaluation [50] of complex interventions. The conceptual definition of acceptability used in this study offers a clear guidance on what experienced acceptability is and what its components are. Without a strong theory base, acceptability is easily confounded with satisfaction [12], thus relying on a framework ensures capturing key dimensions of acceptability. The study sample represents the target group of the original LiFE program [6], is based on clear inclusion criteria from the LiFE-is-LiFE trial (Jansen et al., 2018), and captured varying experiences with the program.

Some limitations need be addressed*.* A larger proportion of women (70%) compared to men were included in our sample. However, this reflects both the population of the trial, with a higher participation rate of women (73.5%), and the general population in older age groups (> 80 years old) with a predominant female demographic [51]. Education level was higher in the focus group sample (53% of participants with German university entrance qualification) compared to the trial (35.7%), as this was not a sampling criterion. We acknowledge that the experiences might have been different for older adults of other background or gender, which could be the focus of future research. Our purposive sampling strategy allowed us to study the groups of older adults most likely to enroll in fall prevention programs like gLiFE or LiFE.

Although free conversation about topics that were relevant to participants was encouraged, the discussion of life experiences or circumstances outside the program that may have influenced the individual’s attitudes towards the programs may have fallen short. As participants lived in the same areas, we could not avoid that some of them were familiar with each other. Overall, participants reported more positive elements about both interventions than negatives. Social desirability, defined as a tendency to reflect reality in a sense that it is consistent with what is perceived as being socially acceptable [52], is a common problem in qualitative research. During focus group discussions, participants were frequently reminded that all answers are acceptable and open discussion was facilitated. Nevertheless, social desirability could have prompted positive rather than negative answers or created a consensus in the group about exercise behavior or opinions on the program [53].

## Conclusion and implications

This is the first study to explore participants’ views on and experiences with gLiFE and LiFE, which are essential factors for the programs’ long-term success [11]. Assessing acceptability yields important information for program development and evaluation and should therefore more often be a component of intervention evaluation. The identified strengths and limitations of both programs from the participants’ perspective could be helpful for program refinement and complement quantitative findings in later stages of the program evaluation. Future studies should focus on possible solutions to the identified limitations, for example the revision of the strategies used in gLiFE to help participants find situations for the implementation of LiFE activities.

In summary, our study showed that fall prone older adults perceived participation in gLiFE and LiFE as beneficial and that both formats were well accepted. Hence, gLiFE and LiFE could be appropriate for implementation within public health initiatives. Whether gLiFE has non-inferior or superior effectiveness compared to LiFE is currently examined [10].

## Supplementary Information

**Additional file 1.** Interview guides of focus group discussions.

## Data Availability

The data used and/or analyzed during the current study are available from the corresponding author on reasonable request.

## Abbreviations

BCT: Behavior Change Technique
BMI: Body Mass Index
gLiFE: Group-based Lifestyle-integrated Functional Exercise
LiFE: Lifestyle-integrated Functional Exercise
MoCA: Montreal Cognitive Assessment
SRBAI: Self-Report Behavioural Automaticity Index
TUG: Timed Up and Go Test
