# Supplementary material for: Group or individual lifestyle-integrated functional exercise (LiFE)? A qualitative analysis of acceptability
Source: BMC Geriatr. 2021 Feb 1;21:93. doi: 10.1186/s12877-020-01991-0 (PMC7852079; doi:10.1186/s12877-020-01991-0)
Supplement: Supplementary file 1 — Additional file 1. Interview guides of focus group discussions. [file 12877_2020_1991_MOESM1_ESM.docx]

Additional file 1: Interview guides

Semistructured interview guide for gLiFE focus groups

| Topic | Main questions | Prompts |
| --- | --- | --- |
| Opening | 1. Introduction round: Please say your name, age and tell us a bit about your previous experiences with exercise programs or courses similar to LiFE. |  |
|  | 1.1 What were your reasons behind your decision to take part in the LiFE program? | Expectations prior to the course start  Reactions of friends and family members to participation |
| Experience with LiFE | 2. What was your personal experience with the LiFE program? | *Ca. 20 minutes of free discussion before proceeding* |
|  | 2.1 To which extent have you enjoyed taking part in the LiFE program? |  |
|  | 2.2 What has changed since you are practicing LiFE? |  |
| Perceptions of group format | 3. Overall, what are your thoughts on the group sessions? | Organization (Travel to study center, Duration and frequency of sessions)  Procedures (theoretical/practical input, organizational setting)  Trainers (competence, way and quality of teaching, safety)  Materials – *materials provided on table*  Phone calls  What can be improved/changed and what is important and should be kept? |
|  | 3.1 Can you tell us how you felt about learning the LiFE activities together with other persons your age (peers)? | In which way did the group affect you? |
| LiFE in daily life | 4. How was it for you to practice the LiFE activities learnt during the group sessions independently at home? | What has helped you?  Difficulties you encountered? |
|  | 4.1 Can you describe a typical practice situation in your home? |  |
|  | 4.2 To which extent have the LiFE activities become habitual?  (habit = automatic, no need to think about the initiation and execution anymore) | Which exercises have become a habit?  What has helped to build the habit?  Cues (activities linked to daily situation/cue? |
|  | 4.3 Do you think you will continue (some of) the LiFE activities? | If yes, why? If no, why not?  Longterm? |
| Closing | 5. Would you recommend the LiFE program to a friend or family member in your age? | Why or why not? |
|  | 5.1 Do you have any ideas as to how the group-LiFE program could be improved? |  |
|  | 5.2 Do you want to add or ask something before we finish? |  |

Semistructured interview guide for LiFE focus groups

| Topic | Main questions | Prompts |
| --- | --- | --- |
| Opening | 1. Introduction round: Please say your name, age and tell us a bit about your previous experiences with exercise programs or courses similar to LiFE. |  |
|  | 1.1 What were your reasons behind your decision to take part in the LiFE program? | Expectations prior to the course start  Reactions of friends and family members to participation |
| Experience with LiFE | 2. What was your personal experience with the LiFE program? | *Ca. 20 minutes of free discussion before proceeding* |
|  | 2.1 To which extent have you enjoyed taking part in the LiFE program? |  |
|  | 2.2 What has changed since you are practicing LiFE? |  |
| Perceptions of individual format | 3. Overall, what are your thoughts on the individual LiFE format? | Duration and frequency of sessions  Procedures of home visits  (theoretical/practical input, organizational setting)  Trainers (competence, way and quality of teaching, safety)  Materials – *materials provided on table*  Phone calls  What can be improved/changed and what is important and should be kept? |
|  | 3.1 Can you tell us how you felt about learning the LiFE activities in your own home? | Advantages/disadvantages of learning LiFE alone |
| LiFE in daily life | 4. How was it for you to practice the LiFE activities independently at home? | What has helped you?  Difficulties you encountered? |
|  | 4.1 Can you describe a typical practice situation in your home (without the trainer)? |  |
|  | 4.2 To which extent have the LiFE activities become habitual? (habit = automatic, no need to think about the initiation and execution anymore) | Which exercises have become a habit?  What has helped to build the habit?  Cues (activities linked to daily situation/cue?) |
|  | 4.3 Do you think you will continue (some of) the LiFE activities? | If yes, why? If no, why not?  Longterm? |
| Closing | 5. Would you recommend the LiFE program to a friend or family member in your age? | Why or why not? |
|  | 5.1 Do you have any ideas as to how the group-LiFE program could be improved? |  |
|  | 5.2 Do you want to add or ask something before we finish? |  |
